# Phenotypic characterization of acute headache attributed to SARS-CoV-2: An ICHD-3 validation study on 106 hospitalized patients

**DOI:** 10.1177/0333102420965146

**Published:** 2020-11-04

**Authors:** Javier Trigo López, David García-Azorín, Álvaro Planchuelo-Gómez, Cristina García-Iglesias, Carlos Dueñas-Gutiérrez, Ángel L Guerrero

**Affiliations:** 1 Headache Unit, Department of Neurology, Hospital Clínico Universitario de Valladolid, Valladolid, Spain; 2 Institute for Biomedical Research of Salamanca (IBSAL), Salamanca, Spain; 3 Imaging Processing Laboratory, Universidad de Valladolid, Valladolid, Spain; 4 Department of Internal Medicine, Hospital Clínico Universitario de Valladolid, Valladolid, Spain; 5 Department of Medicine, School of Medicine, Universidad de Valladolid, Valladolid, Spain

**Keywords:** COVID-19, nervous system diseases, headache disorders, secondary, migraine disorders, tension-type headache

## Abstract

**Introduction:**

Headache is a common symptom of the severe acute respiratory syndrome coronavirus 2 (SARS-CoV-2) infection. In this study, we aimed to characterize the phenotype of headache attributed to SARS-CoV-2 infection and to test the International Classification of Headache Disorders (ICHD-3) phenotypic criteria for migraine and tension-type headache.

**Methods:**

The study design was a cross-sectional study nested in a cohort. We screened all consecutive patients that were hospitalized and had a positive SARS-CoV-2 test. We included patients that described headache if the headache was not better explained by another ICHD-3 diagnosis. Patients were interviewed by two neurologists.

**Results:**

We screened 580 patients and included 130 (mean age 56 years, 64% female). Headache was the first symptom of the infection in 26% of patients and appeared within 24 hours in 62% of patients. The headache was bilateral in 85%, frontal in 83%, and with pressing quality in 75% of patients. Mean intensity was 7.1, being severe in 64%. Hypersensitivity to stimuli occurred in 57% of patients. ICHD-3 criteria for headache attributed to systemic viral infection were fulfilled by 94% of patients; phenotypic criteria for migraine were fulfilled by 25% of patients, and tension-type headache criteria by 54% of patients.

**Conclusion:**

Headache attributed to SARS-CoV-2 infection in hospitalized patients has severe intensity, frontal predominance and oppressive quality. It occurs early in the course of the disease. Most patients fulfilled ICHD-3 criteria for headache attributed to systemic viral infection; however, the phenotype might resemble migraine in a quarter of cases and tension-type headache in half of the patients.

## Abbreviations

SARS-CoV-2: Severe acute respiratory syndrome coronavirus 2

COVID-19: Coronavirus disease 2019

ICHD-3: International Classification of Headache Disorders

STROBE: Strengthening the reporting of observational studies in epidemiology

ERB: Ethics review board

RT-PCR: Real-time reverse transcription polymerase chain reaction

mRS: Modified Rankin Scale

GP: General practitioner

ARDS: Acute respiratory distress syndrome

NRS: Numeric rating scale

SD: Standard deviation

IQR: Interquartile range

## Introduction

Headache is a common symptom of systemic infections (1–3). Evidence of causation is based on the temporal relation between the headache and the onset, worsening, improvement and/or resolution of the infection (4). The severe acute respiratory syndrome coronavirus 2 (SARS-CoV-2) might manifest with headache (5,6). Proper identification and diagnosis of the novel coronavirus disease 2019 (COVID-19) is crucial, on account of the contagiousness of the virus and the substantial mortality rate (6,7).

Patients with COVID-19 might experience headache due to several reasons, including drugs, systemic complications, hypoxemia, neurological impairment, fever, or viraemia (1–3,8,9). Therefore, it seems important both to analyse the presence of specific features of headache attributed to SARS-CoV-2 and to evaluate if headache can mimic the phenotype of other primary headache disorders. The aims of this study were to characterize the phenotype of headache attributed to SARS-CoV-2 infection and to evaluate whether the clinical phenotype fulfilled the International Classification of Headache Disorders, 3rd edition (ICHD-3) criteria for headache attributed to systemic viral infection, migraine or tension-type headache (TTH).

## Methods

The study design was a cross-sectional study nested in a cohort. We followed the Strengthening in the Reporting of Observational Studies in Epidemiology (STROBE) guidelines (10). The study protocol was approved by the Ethics Review Board (ERB) of the Valladolid East health area (code: PI 20-1738). All patients gave written or verbal informed consent, written consent was waived by the ERB because of the potential risk of contagion. The study was conducted at the Hospital Clínico Universitario, Valladolid, Spain, a tertiary academic public hospital with a reference population of 280,000 patients.

### Eligibility criteria

We included patients that: i) had a confirmed COVID-19 case, confirmed by real-time reverse transcriptase polymerase chain reaction (RT-PCR) assay from a respiratory tract sample and/or by the presence of anti-SARS-CoV-2 IgM+IgA antibodies in patients with clinical symptoms, according to the World Health Organization protocols (further details of the tests are available in supplementary materials) (11,12); ii) described headache during the course of COVID-19; and iii) were hospitalized due to COVID-19. First, we excluded those patients with acute secondary causes, in which the headache was better accounted for by another diagnosis of the ICHD-3 criteria (4). Then, we excluded patients if: i) they expired during the hospitalization; ii) they were unable to describe the headache due to a severe medical condition; iii) they had prior history of dementia or cognitive impairment that made it impossible to precisely describe the headache; iv) they did not respond to the invitation; or v) they declined to participate.

We systematically screened every patient that was admitted from 8 March to 11 April 2020. The source of information was the electronic medical records from primary care, the emergency room and the hospitalization. We interviewed every eligible patient about the presence of headache, and those with a positive response were invited to participate. The interviews were conducted by two neurologists between 21 April and 15 May. Those patients that were still hospitalized at that time were interviewed in person, while the discharged patients were contacted by phone or were scheduled for a consultation.

### Variables

We analysed demographic variables, including age, sex and baseline performance assessed using the modified Rankin Scale (mRS) (13). Prior medical history of patients was assessed, including presence of hypertension, diabetes, a smoking habit, a prior history of cardiac, pulmonary, or neurologic disorders, and a history of cancer or immunocompromised conditions (definitions are available in supplementary materials). Prior history of headache disorders was assessed and classified according to the ICHD-3 (4) criteria by the researchers after interviewing the patients. We inquired whether the prior headache had been diagnosed by the general practitioner (GP), a neurologist, or a headache specialist. We also asked patients about prior episodes of headache developed in temporal relation to other systemic infections.

We assessed whether the diagnosis was based on RT-PCR or serological tests or both. We described the results of chest imaging by either x-ray or computerized tomography. The course of COVID-19 was analysed, categorizing severity into mild disease, pneumonia, severe pneumonia or acute respiratory distress syndrome (ARDS) according to the American Thoracic Society guidelines for community acquired pneumonia (Supplemental Table 1) (14). We analysed the initial presenting symptoms of COVID-19 and the most bothersome symptoms.

Concerning the headache, we assessed the time elapsed between the first COVID-19 symptom and the headache onset, if the headache worsened in parallel with the COVID-19 course, and if the headache resolution conformed with that of the infection. We interrogated patients about topography, including whether the headache was bilateral or unilateral, and whether it was localized in the frontal, temporal, parietal, occipital, cervical, periocular, or vertex territories. We studied the quality of the pain and the intensity on a 0–10 numeric rating scale (NRS) (0: No pain, 10: Worst possible pain). Intensity of pain was graded as mild pain (NRS: 1–3), moderate pain (NRS: 4–6) or severe pain (NRS: 7–10) (4). We asked patients about how disabled they felt because of the headache on a 0–100 NRS (0: no disability, 100: absolute disability). We questioned patients about the presence of clinophilia during the headache, defined as the preference for being in a reclined position at rest. We also analysed if the headache was worsened by walking, head movements, ocular movements, coughing, bending, or sneezing. We asked about the presence of photophobia, phonophobia, osmophobia, nausea, and vomiting.

We evaluated headaches according to the ICHD-3 criteria for 9.2.2, headache attributed to systemic viral infection, in the sample. We also evaluated based on ICHD-3 phenotypic criteria C and D for 1.1, migraine without aura, and phenotypic criteria C and D for 2.1, episodic tension-type headache (4). In the first case, we compared patients with and without prior history of migraine; in the second case, we compared patients with and without prior history of TTH.

### Statistical analysis

We describe qualitative variables as frequencies and percentages and continuous variables as means and standard deviations (SD) if the distribution was normal, or medians and inter-quartile ranges (IQR) if not. To compare patients with and without previous migraine or TTH, we used a chi-squared test, considering 0.05 as the statistical significance threshold after adjusting for multiple comparisons by using the Bonferroni method. We did not estimate sample size *a priori*, as all consecutive patients that fulfilled eligibility criteria during the study period were included. We managed missing data by using complete case analysis. Datasheets are available for other researchers under reasonable request. We used SPSS version 26.0 for statistical analysis and BioRender for figures.

## Results

**Figure 1.**
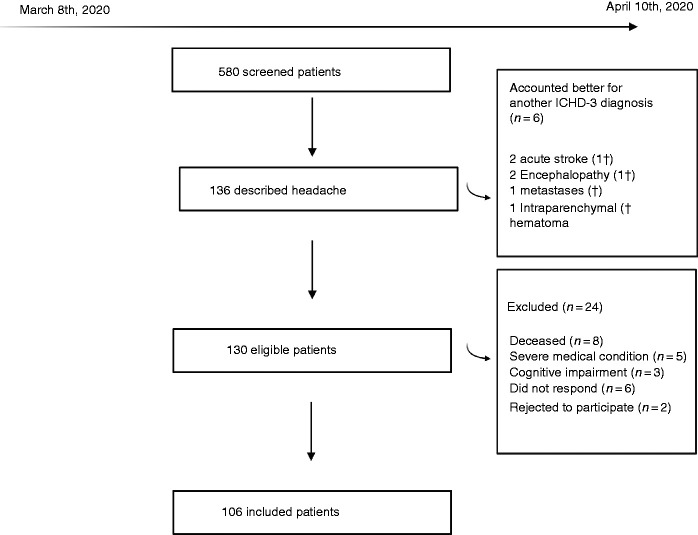
Flow diagram of screened, included and excluded patients, with the reasons for exclusion. † means deceased patient.

During the study period, 580 patients were hospitalized and had a positive COVID-19 test. There were 130 eligible cases that met the inclusion criteria, and the sample included 106 patients after assessing the exclusion criteria. Figure 1 presents the flow diagram with the specific reasons for exclusion.

### Demographics

The mean age of patients was 56.6 (SD: 11.2) years, and 68 (64.2%) patients were female. At baseline, the mean mRS score of patients with headache was 0.09 (SD: 0.40), with a score of 0 in 99 (93.4%) patients, score of 1 in five (4.7%) patients, and a score of 2 and 3 in one (0.9%) single case each. Patients had a prior history of hypertension in 36 (34.0%) cases, diabetes in 12 (11.3%), cardiac disorders in nine (8.5%), pulmonary disorders in 24 (22.6%), cancer in 13 (12.3%), five (4.7%) had an immunocompromised state and 12 (11.3%) were current or former smokers. Prior history of neurological disorders included prior history of stroke in four (3.8%) patients, tremor in two (1.9%) patients, and chronic lower back pain in six (5.7%) patients.

### Prior history of headache

Patients described family history of headache in 40 (37.7%) cases with migraine in 36 (90.0%) of those. Prior history of headache was described by 51 (48.2%) patients. Patients had been diagnosed with headache by their GP in 13 (25.5%) cases, neurologist in 12 (23.5%) cases, or headache specialist in two (3.9%) cases. Of those 51 cases, the specific diagnosis was migraine in 18 cases (17 episodic, one chronic migraine), TTH in 30 cases, cervicogenic headache in three cases, hypnic headache in one case and episodic cluster headache in another case. Two of the patients had both migraine and TTH. The mean frequency of prior headache was 2.1 (SD: 3.9) days per month, being within the episodic range in all cases but one. Prior episodes of headache in temporal relation to the onset of prior infections were described by 50 (47.2%) patients, including six patients with migraine and 20 with TTH. Those patients described that the headache experienced during COVID-19 was similar to headaches suffered during other infections in 23 (45.1%) cases.

### Diagnosis and severity of COVID-19

Diagnosis was based on RT-PCR in 103 cases (97.2%) and IgM+IgA serology in 36 (34.0%). Chest X-ray was abnormal in 101 (95.3%) cases. Degree of severity corresponded to mild disease in five (4.7%) cases, standard pneumonia in 47 (44.3%) cases, severe pneumonia in 46 (43.4%) cases, and ARDS in eight (7.5%) cases. The mean mRS score on discharge was 0.32 (SD: 0.80) and was worse than baseline in 14 (13.2%) patients (Supplemental Table 2 shows the change in mRS from baseline to discharge).

### Headache during the course of the disease

**Figure 2.**
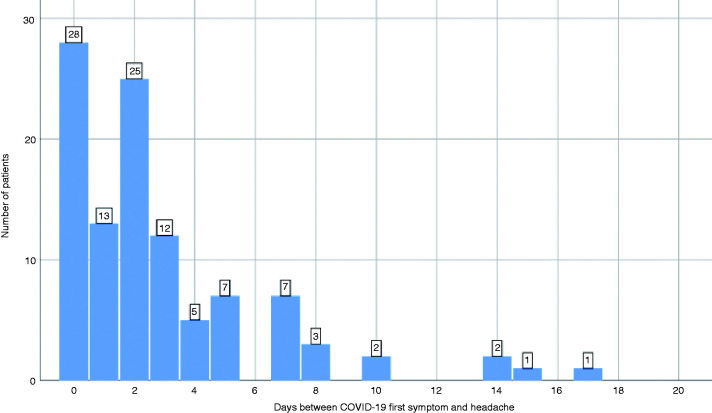
Interval (in days) between the first COVID-19 symptom and the headache onset. Frequency of patients.

The most frequent COVID-19 presenting symptoms were fever in 31 (29.2%), headache in 28 (26.4%), cough in 12 (11.3%), and asthenia in 12 (11.3%) patients. Headache developed within 24 hours of the onset of COVID-19 symptoms in 41 (38.7%), within 48 hours in 66 (62.3%), and within 72 hours in 78 (73.6%) patients. Figure 2 shows the numbers of days between the first COVID-19 symptom and the headache.

Headache worsened in parallel with the worsening of the disease in 42 (39.6%) cases. Headache had resolved in parallel with the improvement or resolution of COVID-19 in 58 (54.7%) cases. The most bothersome COVID-19 symptom was fever for 21 (19.8%) patients, headache for 20 (18.9%), dyspnoea for 20 (18.9%), asthenia for 10 (9.4%) and myalgia for nine (8.5%). By the time the survey was completed, COVID-19 had resolved in 84 (79.2%) patients, with headache persisting in 38 (45.2%) of those 84. Of the remaining 22 (20.8%) patients with active SARS-CoV-2 infection, 10 (45.5%) were still experiencing headache at the time of the interview.

### Headache phenotype

**Figure 3.**
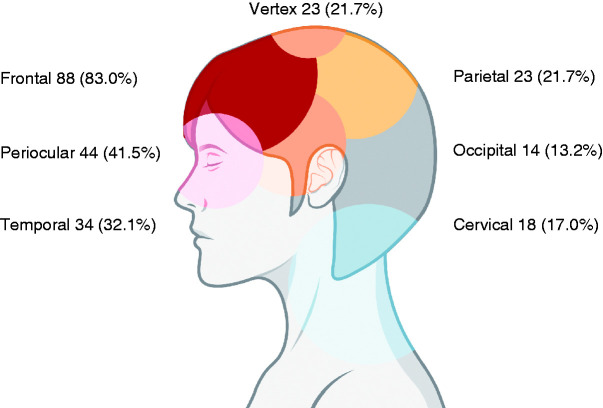
Topography of the headache. Number of patients that described pain in each topography.

The topography of the headache was bilateral in 90 patients (84.9%) and hemicranial in 16 (15.1%). The most frequent topographies of the headache were frontal in 88 (83.0%) patients, periocular in 44 (41.5%) patients, and temporal in 34 (32.1%) patients. Figure 3 shows the number of patients that described pain in each topographical region. Pain was circumscribed within one of those areas in 35 (33.0%) patients, two of those areas in 43 (40.6%), three areas in 14 (13.2%), or four areas in six (5.7%) and was described as holocranial by the remaining eight (7.5%) patients. The pain affected two or more areas and was bilateral in 58 (54.7%) of cases.

**Figure 4.**
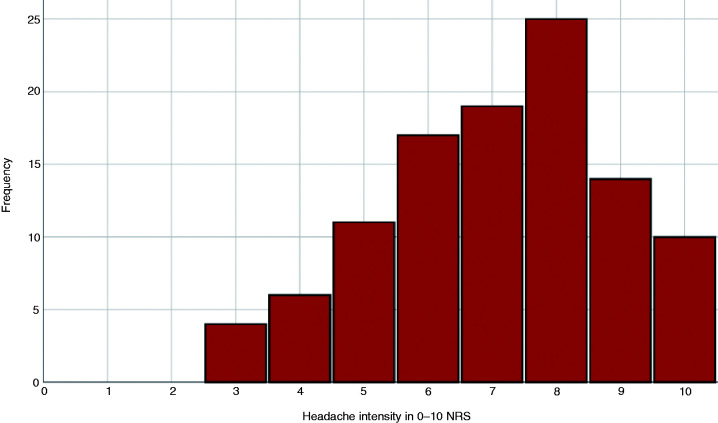
Intensity of the headache on a 0–10 numeric rating scale (NRS).

**Figure 5.**
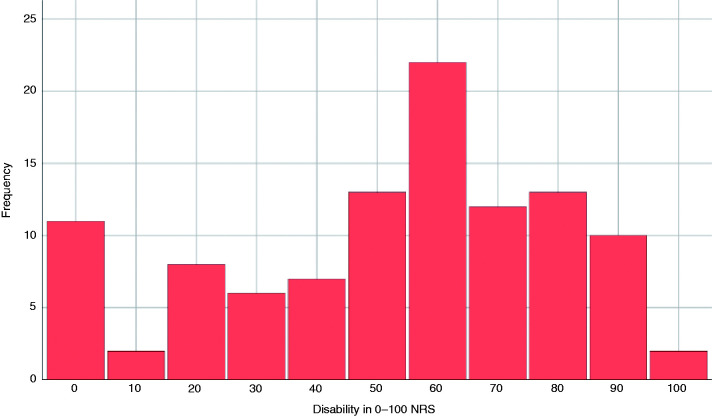
Degree of disability on a 0–100 numeric rating scale (NRS).

The quality of the pain was pressing in 80 (75.5%) patients, pulsating in 23 (21.7%), stabbing in 15 (14.2%), burning in three (2.8%), and electric in one (0.9%). The mean intensity of the headache was 7.1 (SD: 1.8), being mild in four (3.8%) cases, moderate in 34 (32.1%) and severe in 68 (64.1%). Figure 4 shows the intensity of the headache according to the score on the 0–10 NRS. The median degree of disability caused by the headache was 60 (IQR: 30–70). Figure 5 depicts the level of disability on a 0–100 NRS. Clinophilia was described by 61 (57.5%) patients.

The headache was worsened by walking in 11 (10.4%), with movement of the head in 33 (31.1%) cases, and with movement of the eyes in 20 (18.9%) patients. The headache worsened with coughing in 35 (33.0%) patients, by bending in seven (6.6%) patients, and by sneezing in two (1.8%) patients.

Hypersensitivity to stimuli was described by 60 (56.6%) patients, with photophobia in 48 (45.3%) patients, phonophobia in 42 (39.6%) and osmophobia in two (1.8%). Nausea and/or vomiting was described by 15 (14.2%) patients.

### Evaluation of ICHD-3 criteria

All participants included in the study fulfilled criteria B and D for 9.2.2, headache attributed to systemic viral infection. Criterion C was fulfilled by 100 (94.3%) of the participants. The least frequently fulfilled criteria were C.4.a (diffuse pain) and C.2 (worsening in parallel with the worsening of the infection). Table 1 shows the number of patients that fulfilled each criterion.

**Table 1.** ICHD-3 criteria for 9.2.2 headache attributed to systemic viral infection.

Criterion	Frequency
A. Headache of any duration fulfilling criterion C	
B. Both of the following:	106 (100%)
1. Systemic viral infection has been diagnosed	106 (100%)
2. No evidence of meningitic or encephalitic involvement	106 (100%)
C. Evidence of causation demonstrated by at least two of the following:	100 (94.3%)
1. Headache has developed in temporal relation to onset of the systemic viral infection	78 (73.6%)
2. Headache has significantly worsened in parallel with worsening of the systemic viral infection	42 (39.6%)
3. Headache has significantly improved or resolved in parallel with improvement or resolution of the systemic viral infection	58 (54.7%)
4. Headache has either or both of the following characteristics:	(99.1%)
4a. Diffuse pain	(54.7%)
4b. Moderate or severe intensity	102 (96.2%)
D. Not better accounted for by another ICHD-3 diagnosis	106 (100%)

ICHD-3: The International Classification of Headache Disorders, 3rd edition.

Phenotypic criteria for 1.1, migraine without aura, were fulfilled by 55 (51.9%) of the patients for criterion C and by 40 (37.7%) patients for criterion D. Patients with prior history of migraine more frequently had unilateral headache (p = 0.004). Both criteria C and D were fulfilled by 27 (25.5%) patients, including seven (38.9%) patients with prior history of migraine and 20 (22.7%) patients with no migraine history (p = 0.256). Table 2 shows the number of patients that fulfilled ICHD-3 phenotypic criteria for migraine.

**Table 2.** Phenotypic ICHD-3 criteria C and D for 1.1 migraine without aura.

Criterion	All patients (n = 106)	No history of migraine (n = 88)	Prior history of migraine (n = 18)	Adjusted *p*-value
C. Headache has at least two of the following four characteristics				
Unilateral location	16 (15.1%)	8 (9.1%)	8 (44.4%)	0.004
Pulsating quality	23 (21.7%)	20 (22.7%)	3 (16.7%)	1
Moderate or severe pain intensity	102 (96.2%)	84 (95.5%)	18 (100%)	1
Aggravation by or causing avoidance of routine physical activity	36 (34.0%)	25 (28.4%)	11 (61.1%)	0.133
At least two:	55 (51.9%)	43 (48.9%)	12 (66.7%)	1
During the headache at least one of the following				
Nausea and/or vomiting	15 (14.2%)	12 (13.6%)	3 (16.7%)	1.000
Photophobia and phonophobia	31 (29.2%)	23 (26.1%)	8 (44.4%)	1
At least one	40 (37.7%)	31 (25.2%)	9 (50%)	1

ICHD-3: The International Classification of Headache Disorders, 3rd edition.

Phenotypic criterion C for 2.1, episodic tension-type headache, was fulfilled by 90 (84.9%) of the patients and phenotypic criterion D by 66 (62.3%) of the patients. Those criteria were not more frequently fulfilled in patients with prior history of TTH (p > 0.1 in all cases). Both criteria were fulfilled by 57 (53.8%) patients, including 13 (43.3%) patients with prior TTH and 44 (57.9%) with no TTH history (p = 0.255). There were 39/106 (36.7%) patients that fulfilled both criterion C for migraine and criterion C for TTH, and no patients fulfilled criterion D for both disorders; therefore, there were no patients that fulfilled both ICHD criteria C-D for migraine and TTH. Table 3 presents the number of patients that fulfilled ICHD-3 phenotypic criteria for TTH. Of the patients who had prior history of migraine or TTH, we did not observe differences in the frequencies of persistent or non-persistent headache, but patients with pre-existing headache showed a trend to fulfil more ICHD-3 criteria for migraine (p = 0.033; the statistical significance threshold was 0.006 after adjusting for multiple comparisons). The full details of the comparison are presented in Table 4.

**Table 3.** Phenotypic ICHD-3 criteria C and D for 2.1 episodic tension-type headache.

	All patients (n = 106)	No history of TTH (n = 76)	Prior history of TTH (n = 30)	Unadjusted *p*-value
Criterion C: Headache has at least two of the following four characteristics				
Bilateral location	90 (84.9%)	64 (84.2%)	26 (86.7%)	0.986
Pressing or tightening	80 (75.5%)	58 (72.5%)	22 (73.3%)	0.943
Mild or moderate intensity	38 (35.8%)	28 (36.8%)	10 (33.3%)	0.909
Not aggravated by routine physical activity	70 (66.0%)	51 (67.1%)	19 (63.3%)	0.887
At least two:	90 (84.9%)	66 (86.8%)	24 (80.0%)	0.558
Criterion D: During the headache at least one of the following				
No nausea or vomiting	91 (85.8%)	64 (84.2%)	27 (90.0%)	0.645
No more than one of photophobia or phonophobia	75 (70.8%)	57 (75.0%)	18 (60.0%)	0.196
Both of them	66 (62.3%)	50 (65.8%)	16 (53.3%)	0.332

ICHD-3: The International Classification of Headache Disorders, 3rd edition; TTH: tension-type headache.

**Table 4.** Prior history of headache and headache phenotype in patients with persistent headache.

	All patients (n = 106)	Persistent headache (n = 48)	Non-persistent headache (n = 58)	Unadjusted *p*-value
Prior history of migraine	18 (17.0%)	10 (20.8%)	8 (13.8%)	0.337
Criterion C for migraine	55 (51.9%)	28 (58.3%)	27 (46.6%)	0.227
Criterion D for migraine	40 (37.7%)	22 (45.8%)	18 (31.0%)	0.118
Both criteria C and D for migraine	27 (25.5%)	17 (35.4%)	10 (17.2%)	0.033
Prior history of TTH	30 (28.3%)	13 (27.1%)	17 (29.3%)	0.800
Criterion C for TTH	90 (84.9%)	40 (83.3%)	50 (86.2%)	0.787
Criterion D for TTH	66 (62.3%)	26 (54.2%)	40 (69.0%)	0.118
Criteria C and D for TTH	57 (53.8%)	24 (50%)	33 (56.9%)	0.478

TTH: tension-type headache.

## Discussion

Diagnosis and classification of headache disorders should always consider the entire differential diagnosis. All of the specific headache diagnoses in the ICHD-3 require that the headache is not better accounted for by another ICHD-3 diagnosis (4). In this study, we depicted the phenotype of headache attributed to SARS-CoV-2 infection and analysed if the headache phenotype mimicked migraine or TTH. We focused on those two primary headache disorders because of their high prevalence (15), and we elected to study phenotypes here considering that some clinicians focus on phenotype instead of concentrating on the temporal course of the headache or the presence of red flags (16).

COVID-19 is a life-threatening condition. In Spain, the mortality rate for 250,287 reported cases was 8.2%, with death occurring after a median of 11 days (IQR 7–17). The mortality rate increased to 18% in patients with pneumonia and to 31% in patients that were admitted to the intensive care unit (7). This mortality rate is comparable to other life-threatening secondary headache disorders, such as cerebral venous sinus thrombosis (12%) (17), central nervous system infections (23%) (18) or intracranial haemorrhage (35%) (19). In the case of COVID-19, early diagnosis is important not only for the treatment of the patients, but also for avoiding the transmission of the disease (6,12).

Confirmation of COVID-19 is based on microbiological tests, either a positive RT-PCR test or the presence of IgM antibodies in patients with typical clinical symptoms (11,12). During the pandemic, every patient should be treated as a potential case (11). However, microbiological tests are not always available, and therefore clinicians must be vigilant of the possibility of COVID-19 in patients complaining of headache (20). Literature describing the headache phenotype of viral infections is scarce (1,2), and in the case of COVID-19, unexplored (8).

Headache is a frequent symptom in systemic infections. Up to 60% of patients with an upper respiratory tract infection describe headache (21). Headache is a common symptom in patients with viral infection, described in 47–55% of patients with influenza (22), 17% of patients with Epstein-Barr virus infectious mononucleosis, and 48% of patients with cytomegalo virus infectious mononucleosis (23). Headache is also frequently reported in other diseases such as malaria (50–75%) (24,25) and dengue (59.4%) (26). The pathophysiology of the headache in systemic infections might be linked to cytokine release (27), the presence of fever (1), systemic inflammation (1,2), or the direct damage caused by the pathogens (2), among other factors.

Diagnosis of secondary headache disorders is still based on the presence of red and orange flags (28,29). Most of the proposals for these red and orange flags include items such as fever, the presence of systemic symptoms, recent onset, or progressive worsening, and these signs may also be helpful in the case of COVID-19 (28). Despite the fact that many patients might describe a headache phenotype similar to migraine or TTH, the presence of a single red flag must obligate clinicians to rule out a secondary cause (4,16,28). In addition, despite the TTH phenotype being more frequent than the migraine-like phenotype, a third of patients in this study had both migraine and TTH phenotypic features. It is possible that the pathophysiology of headache in COVID-19 patients might be closer to migraine than TTH. The reason why some patients experience a persistent headache is still unclear. Future studies should follow up with those patients and clarify why headache persists in some cases. Indeed, in our sample, patients with persistent headache exhibited migraine-like features more frequently than those patients in which the headache resolved.

Headache is a disabling symptom of COVID-19. Pain was described as severe by 64% of patients and was the most bothersome symptom of COVID-19 for 19% of the patients, and the median disability was estimated to be 60 out of 100. Aside from the proper diagnosis and treatment of COVID-19, symptomatic treatment of the headache seems necessary. The use of some of the available acute headache medications has been controversial (30), including non-steroidal anti-inflammatory drugs or steroids; evidence suggesting that they might be harmful is inconclusive (30,31).

The present study has important limitations. We selected hospitalized patients because, at the beginning of the pandemic, the shortage of personal protective equipment and diagnostic tests prioritised the use of RT-PCR for those patients that needed admission. Future studies should analyse if the headache phenotype in an outpatient setting is similar and if higher severity of COVID-19 is associated with a more severe headache. At the other end of the spectrum, we could not evaluate the most severe patients because they were deceased or in critical condition by the time the study was done. We were unable to reach 24/130 (18.4%) patients, which could indicate a selection bias. Evaluation of the patients was cross-sectional, so we were not able to properly differentiate if patients developed different headache phenotypes throughout the course of the disease, and some patients might have been subject to recall bias. We encourage future researchers to design prospective studies to resolve this limitation, systematically assessing patients beginning with their admission and continuing through to the disease resolution. The present study was a single-centre study, which could affect the external validity of the results. Future studies should consider multicentric and even multinational designs. To assess the specificity of the clinical features of headache attributed to COVID-19, future studies should consider control groups with headaches occurring during other viral infections.

## Conclusion

Headache attributed to SARS-CoV-2 infection in hospitalized patients is typically a headache of moderate to severe intensity with frontal predominance and oppressive quality. Headache develops within 72 hours of COVID-19 onset in most cases and might be the presenting symptom in a quarter of patients. Almost all patients in this study fulfilled ICHD-3 criteria for headache attributed to systemic viral infection with the least frequently fulfilled criterion being the diffuse topography of pain. Clinicians should be aware of COVID-19 in all headache differentials, as phenotypic features of migraine and/or TTH were common.

## Clinical implications

- Headache attributed to SARS-CoV-2 infection fulfilled ICHD-3 criteria in most cases.
- Half of the patients with headache and confirmed SARS-CoV-2 infection described a headache phenotype that mimicked TTH, and between a quarter and half of patients described a migraine-like headache, regardless of the prior history of those disorders.
- Headache is a disabling symptom of COVID-19, being the most bothersome symptom for 19% of the patients, having severe intensity in 64% of the patients, and causing a median disability of 60 out of 100 in this study.

## Supplemental Material

sj-pdf-1-cep-10.1177_0333102420965146 - Supplemental material for Phenotypic characterization of acute headache attributed to SARS-CoV-2: An ICHD-3 validation study on 106 hospitalized patients Click here for additional data file. Supplemental material, sj-pdf-1-cep-10.1177_0333102420965146 for Phenotypic characterization of acute headache attributed to SARS-CoV-2: An ICHD-3 validation study on 106 hospitalized patients by Javier Trigo López, David García-Azorín, Álvaro Planchuelo-Gómez, Cristina García-Iglesias, Carlos Dueñas-Gutiérrez and Ángel L Guerrero in Cephalalgia
