# Supplementary material for: Phenotypic characterization of acute headache attributed to SARS-CoV-2: An ICHD-3 validation study on 106 hospitalized patients
Source: Cephalalgia. 2020 Nov 4;40(13):1432–42. doi: 10.1177/0333102420965146 (PMC7645601; doi:10.1177/0333102420965146)
Supplement: sj-pdf-1-cep-10.1177_0333102420965146 - Supplemental material for Phenotypic characterization of acute headache attributed to SARS-CoV-2: An ICHD-3 validation study on 106 hospitalized patients [file sj-pdf-1-cep-10.1177_0333102420965146.pdf]

Supplementary materials:

Diagnosis of Covid-19 disease:

The diagnosed was done in the presence of positive results of real-time reverse-transcriptase-polymerase-chain-reaction (RT-PCR) assay (LightMIx Modular SARS-CoV (COVID19) E-gene and LightMIx Modular SARS-CoV (COVID19) RdRP, Roche Diagnostics S.L.) from oropharyngeal-nasopharyngeal swab, sputum or lower respiratory tract sample; or by the presence of anti-SARS-CoV-2 IgM+IgA antibodies (COVID-19 ELISA IgM+IgA; Vircell, S.L. Granada, Spain) in serological test in patients with clinical symptoms.

References:

1. World Health Organization. Coronavirus diseaase (COVID-19) technical guidance: laboratory testing for 2019-nCoV in humans https://www.who.int/emergencies/ diseases/novel-coronavirus-2019/technical -guidance/laboratory-guidance. Accessed May 31st, 2020.
2. Ministry of Health. Technical documents. Hospital management of COVID-19. Version March 19, 2020. https://www.mscbs.gob.es/profesionales/saludPublica/ccayes/alertasActual/nCov-China/documentos/Protocolo_manejo_clinico_ah_COVID-19.pdf. Accessed on May 31st, 2020.

Variable definitions:

We analyzed variables according to the following definitions:

1. Arterial hypertension as: systemic blood pressure higher than 140/90 mmHg in two prior determinations.
2. Diabetes as: fasting blood glucose >126 mg/dl on two separate tests, HbA1c > 6,5%, blood glucose level >200 mg after oral glucose overload or blood glucose level >200 mg/dl with diabetes symptoms.
3. Smoking habit if patients were smoking in the present or discontinued it in the preceding 6 months.
4. Cardiovascular diseases, including coronary artery disease, congenital heart diseases, cardiomyopathies, arrythmias, valvular heart disease, aortic aneurysms and peripheral artery disease.

5. Chronic pulmonary diseases, including chronic obstructive pulmonary disease (COPD), asthma, occupational lung diseases, interstitial lung diseases and pulmonary hypertension.
6. History of cancer, excluding cutaneous epidermoid and basal cell carcinoma.
7. Immunocompromised state, either congenital or acquired.
8. Prior history of neurological disorders.

Supplemental table 1

Severity of Covid-19 disease according to the American Thoracic Society guidelines for community-acquired pneumonia.

Severity	Description
Mild illness	Patients with uncomplicated upper respiratory tract viral infection symptoms and have non-specific symptoms such as fever, fatigue, cough (with or without sputum production), anorexia, malaise, muscle pain, sore throat, dyspnea, nasal congestion, diarrhea, nausea or vomiting or
Pneumonia	Presence of pneumonia but no signs of severe pneumonia and no need for supplemental oxygen.<br><br>CURB scale≤1.
Severe pneumonia	Confirmed respiratory infection, plus one of the following:<br><br>1) Respiratory rate > 30 breaths/min.<br>2) Severe respiratory distress.<br>3) SpO2 ≤ 93% on room air.
Acute respiratory distress syndrome (ARDS)[16]	**Onset:** within 1 week of a known clinical insult or new or worsening respiratory symptoms.<br><br>**Chest imaging** (radiograph, CT scan, or lung ultrasound): bilateral opacities, not fully explained by volume overload, lobar or lung collapse, or nodules.<br><br>**Origin of pulmonary infiltrates**: respiratory failure not fully explained by cardiac failure or fluid overload. Need

	objective assessment (e.g. echocardiography) to exclude hydrostatic cause of infiltrates/oedema if no risk factor present.<br>**Oxygenation impairment in adults**:<br><br>• Mild ARDS: 200 mmHg < PaO2/FiO2[a] ≤ 300 mmHg (with PEEP or CPAP ≥ 5 cmH2O, or non-ventilated)<br>• Moderate ARDS: 100 mmHg < PaO2/FiO2 ≤ 200 mmHg (with PEEP ≥ 5 cmH2O, or non-ventilated)<br>• Severe ARDS: PaO2/FiO2 ≤ 100 mmHg (with PEEP ≥ 5 cmH2O, or non-ventilated)<br>• When PaO2 is not available, SpO2/FiO2 ≤ 315 implies ARDS (including in non-ventilated patients).

Sp: Saturation percentage. ADRS: Acute Distress Respiratory Syndrome. CT: Cranial Tomography. PaO2: Partial pressure of Oxygen. FiO2: Fraction of inspired Oxygen. PEEP: Positive end-expiratory pressure. CPAP: Continuous positive airway pressure.

Reference:

1. Metlay JP, Waterer GW, Long AC, et al. Diagnosis and treatment of adults with community-acquired pneumonia: an official clinical practice guideline of the American Thoracic Society and Infectious Disease Society of America. *Am J Respir Crit Care Med* 2019;200(7):e45-e67.

Supplemental table 2

Rankin score at the baseline and at discharge:

	Baseline	Discharge
Mean score (SD)	0.09 (0.40)	0.32 (0.80)
Median score (IQR)	0 (0-0)	0 (0-0)
Number of patients with per each category		
0 points	99 (93.4%)	86 (81.1%)
1 point	5 (4.7%)	10 (9.4%)
2 points	1 (0.9%)	4 (3.8%)
3 points	1 (0.9%)	4 (3.8%)
4 points	0 (0%)	1 (0.9%)
Missing data	0	1 (0.9%)
